# American Medical Association

**Published:** 1897-07

**Authors:** 


### AMERICAN MEDICAL ASSOCIATION.

The forty-eighth annual meeting was held in Philadelphia June 1st–4th, 1897, inclusive. Dr. Nicholas Senn, of Chicago, President, and Dr. Wm. Atkinson, Philadelphia, Secretary, in their places.

Addresses of welcome were delivered by the Mayor of Philadelphia, the Hon. Charles F. Warwick, and by the Hon. Chas. Emory Smith for Governor Hastings on the part of the State, the Governor being detained by duties in Harrisburg.

The President's Address, by Dr. Senn, related to "The American Medical Association—Its Past, Its Present, and Its Future." It is especially appropriate to hold this semi-centennial session in Philadelphia—the birthplace of the Association just fifty years ago—whose citizens, beginning with Benjamin Rush, one of the signers of the Declaration of Independence, had always been loyal to the cause of medicine, and which city has so long remained the center of medical education. Drs. N. S. Davis, of Chicago, Alfred Stille, of Philadelphia, J. B. Johnson, of St. Louis, and D. F. Atwalter, of Springfield, Mass., are perhaps the only surviving men who were most helpful in laying the corner-stone of this National Association.

Among reforms brought about by this Association, he remarked that nearly all medical schools now require a four years' course and an attendance of eight months in the year. This reform places our medical schools on a par with those of the old world. He predicted that within twenty-five years more, this country would become the center of medical education; and that instead of going to Europe to complete medical education, European students would be forced to come to America to perfect themselves in the science and art of medicine. While polyclinics and postgraduate medical schools were doing excellent work, they never could or would take the place of medical societies. One who had been living out of reach with his fellows might be benefited by attending these polyclinics; but rational postgraduate work consists in reading current medical literature, in mastering modern text-books, in the use of the microscope, in interpreting disease from a bacteriologic standpoint, and in regular attendance of the meetings of medical societies—local, State and national. So that every recent graduate should affiliate with medical societies. The two apparent dangers of medical societies now are: One, too many societies; and, two, too many papers. The organization of district and sectional medical societies he thinks of questionable utility, as they tend to detract from local, State and national associations. Political doctors, wire-pullers, etc., who seldom read papers or take part in scientific discussions, are the worries of medical societies. The young graduate should listen and say little in his local society for a year or two; in five years he was ready for service in his State society; and when he had ten or twelve years of experience, if he had something valuable to say, he was ready to take part in the national association. All physicians should attend their societies solely to receive or impart information. Dr. Senn urged the creation of a fund for prize essays. He urged the contribution of funds sufficient to erect the Rush monument. He suggested that during the meetings of the Association, lectures should be given on bacteriology, histology, etc., illustrated fully. He thought that there should be an Association dinner each session, to be paid for by the members who participate. He thinks there is need of a permanent home for the Association, an editorial office, press-room, a circulating library, etc. The *Journal* of the Association has passed the trial stage and will some day rival the *British Medical Journal.*

Dr. A. L. Gihon, for the Committee on the Rush Monument Fund, reported that, while the mass of the regular profession in America had succeeded in twelve years in raising only $4,000, the small body of homœopaths in this country had in four years raised $75,000 and erected a monument to Hahnemann. The report stirred up the members, and $2,000 were at once subscribed, and promises of much more from various States, etc.

An Executive Council of five, consisting of three officers of the Executive Committee and two officers to be elected, was decided on, which shall transact the duties commonly assigned to the Executive Committee during the intervals between the meetings. One of the five Councilmen shall belong to the Section on Practice and one to the Section on Surgery and Anatomy.

A further amendment to the Constitution adopted reads: "Any State or local medical society, or other organized institution, whose rules, regulations and code of ethics agree in principle with those of this Association, may be entitled to representation on the advice or agreement of the judicial council.

SECOND DAY.

An invitation from the Atlantic City (New Jersey) Medical Society was received, to become their guests, on Saturday, June 5th.

The Address in Medicine was delivered by Dr. Austin Flint, of New York. Never, since medicine became a science, has medical history been made so fast as now. He hoped to see, in early years of the second half century of this Association, a more complete unity of the profession through its authority and influence. Uniformity of legal qualifications to practice medicine in different States could best be secured by making every State Society actually, as well as nominally, a branch of the American Medical Association, with permanent committees from each State organization to constitute a central legislative body, the object of which should be to secure uniform medical laws in all the States, making any State license valid for all, and a matriculation certificate for one State good for matriculation in all schools represented in the Association of American Medical Colleges. The remainder of his address related to Stercorin and Cholesteræmia.

The report of the Executive Committee showed that about 2,000 members were in attendance upon this session—the largest number ever recorded as present at any one meeting.

To expedite registration of members at future sessions, it was resolved that the Secretary of the Association be constituted a committee of one to conduct in the city where a meeting is to be held, for three days prior to the commencement of the meeting, a bureau of registration for the attending delegates, for which services he is to receive $100 annually.

President McKinley and Governor Hastings were introduced; made brief congratulatory addresses and were cheered to the echo.

Protest against anti-vivisection legislation.—Dr. Horatio C. Wood, of Philadelphia, introduced a resolution, unanimously adopted, protesting against the passage by Congress of Senate bill, No. 1,061, intended to prevent vivisection in the District of Columbia.

THIRD DAY.

A resolution recommending the formation of a National Medical Aid Association was, on motion, referred to a committee of one from each State to establish such an association.

The Board of Trustees was directed to secure a charter, so that the association may legally sue or be sued. There was a cash balance of $7,160 in the treasury. It was recommended that no change in the *Journal* office from Chicago be made. A tribute was paid the editorial staff.

Jubilee Proceedings.—After the address in Surgery by Dr. W. W. Keen, Dr. N. S. Davis, of Chicago, the founder of the association, made an address on the Origin and History of the American Medical Association.

Dr. Geo. Ben. Johnston, of Richmond, President Medical Society of Virginia, spoke for the Presidents of the State Medical Societies. To-day, every State had its Medical Society, modelled after the general plan, and with common purposes in view. Through these Societies there are annually made scores of valuable contributions—these Societies thus acting as great educators, producing and diffusing useful knowledge. A valuable result of legislation begun by these State Societies is the establishment of State Boards of Examiners in nearly every State. By legislation secured through these societies, the self-constituted and empiric are fast passing away, and their places are being filled by educated, competent, and intelligent doctors. Careless and inferior schools soon saw their graduates excluded by the State Boards of Examiners from the privilege of practice, and their classes diminished—the effect of which has been the elevation of the standard of medical education. The National Confederation of State Medical and Licensing Boards was a natural outgrowth; and this confederation must become the leader in uniform laws regulating the practice of medicine. Through State Societies also, State Boards of Health had been established, and are of inestimable value to the public in promulgating hygienic laws, in the prevention and suppression of epidemics, and in the conduct of commerce in times of pestilential danger.

Dr. Wm. Perry Watson, of New Jersey, followed with an address on State Boards of Medical Examiners: What they have done, how they should be made up, and what they should do.

Officers, etc., elected for 1897-8: President—Dr. Geo. M. Sternberg, Surgeon-general, U. S. Navy, Washington, D. C. Vice-Presidents—Drs. Joseph M. Matthews, Louisville, Ky.; J. L. Thompson, Indianapolis, Ind,; F. H. Wiggin, New York; T. J. Happell, Nashville Tenn. Secretary—Dr. Wm. B. Atkinson, Philadelphia, Pa. Treasurer—Dr. H. P. Newman, Chicago, Ill. Librarian—Dr. Geo. W. Webster, Chicago, Ill. Board of Trustees—terms to expire 1900, Drs. Joseph Eastman, Indianapolis; J. F. Priestley,———, Iowa; and Truman W. Miller, ———, Ill. Judicial Council, terms to expire 1900.—All former members re-elected. To deliver Address on Medicine, 1898—Dr. J. H. Masser, Philadelphia, Pa. Address on Surgery, 1898—Dr. John B. Murphy, Chicago, Ill. Address on State Medicine, 1898—Dr. Samuel C. Busey, Washington, D. C. Place of

Meeting, May 1898—Denver, Colorado. Chairman of Committee of arrangements, Dr. J. W. Graham, Denver.

Resolutions of sorrow were adopted because of the death of Dr. Jerome Cochran, late health officer of Alabama.

FOURTH DAY.

Delegates to International Medical Congress at Moscow, August, 1897, were elected.

The address on State Medicine was delivered by Dr. John B. Hamilton, his subject being mostly limited to the prevention of tuberculosis.

Resolutions of thanks were voted the Philadelphia doctors and citizens generally for their contribution to the most uniformly successful session of the Association.

After introducing the president-elect, Dr. Sternberg, the session adjourned *sine die.—Virginia Medical Semi-Monthly.*

